# Supplementary material for: Social support and physical exercise: The mediating role of achievement emotions, and the moderating role of exercise motivation and feedback literacy
Source: PLoS One. 2025 Jun 2;20(6):e0325365. doi: 10.1371/journal.pone.0325365 (PMC12129193; doi:10.1371/journal.pone.0325365)
Supplement: Attachment — Submitted filename: Response to Reviewers.doc [file pone.0325365.s002.doc]

**Rebuttal letter**

Dear editor/reviewers：

Thank you for your constructive feedback and the opportunity to revise our manuscript. We have carefully addressed all comments and made corresponding revisions to the manuscript. Below is our point-by-point response to the reviewers’ concerns.

**1. PLOS ONE's style Statement**

Comment: Please ensure that your manuscript meets PLOS ONE's style requirements.

Response:

We have revised the manuscript according to the style requirements of PLOS ONE, please review it.

**2. Role of Funder Statement**

Comment: Please state the role of the funders in the study.

Response:

As requested, we have added the following statement in the cover letter:

The funders had no role in study design, data collection and analysis, decision to publish, or preparation of the manuscript.

**3. Data Availability Statement**

Comment: Confirm whether all raw data required to replicate the results are provided.

Response:

Raw data for all statistical analyses are provided in S1 Achievement emotion in social support and physical exercise Dataset.

<https://datadryad.org/dataset/doi:10.5061/dryad.s7h44j1jh>

### **4. Ethics Statement**

Comment: Provide full ethics committee details and informed consent information.

Response:

We have added the following ethics statement to the “Methods” section:

This study was approved by the Project Approval Committee Ethics Approval Panel. Written informed consent was obtained from all participants prior to data collection.

Change in manuscript: See “Methods” section (Page 5, Line 104-105).

5. **Reference List**

Comment: Ensure references are complete and avoid citing retracted articles.

Response:

We have thoroughly reviewed the reference list and removed citations to any retracted articles. Verified the accuracy of all references using Google Scholar. Ensured that all cited works are formatted according to the journal’s guidelines.

We have changed references 1, 6 and 9. Because they can only look it up in China National Knowledge Infrastructure, not in Google Scholar.

Change in manuscript: Updated references are marked in the revised manuscript (Pages 19–21).

6. **Participants**

We added Hubei Enshi University in the Participants section, because we found that it was omitted when we edited the manuscript.

Change in manuscript: See “Participants” section (Page 5, Line 101).

We sincerely appreciate your time and consideration. Please contact us if further revisions are required.

Best regards,

Zhang Hui

On behalf of all authors
