# Supplementary material for: Social support and physical exercise: The mediating role of achievement emotions, and the moderating role of exercise motivation and feedback literacy
Source: PLoS One. 2025 Jun 2;20(6):e0325365. doi: 10.1371/journal.pone.0325365 (PMC12129193; doi:10.1371/journal.pone.0325365)
Supplement: Attachment — Submitted filename: Response_to_Reviewers_auresp_2.doc [file pone.0325365.s003.doc]

**Rebuttal letter**

Dear editor/reviewers：

Thank you for your constructive feedback and the opportunity to revise our manuscript. We have carefully addressed all comments and made corresponding revisions to the manuscript. Below is our point-by-point response to the reviewers’ concerns.

**Responses to Reviewer #2**

**1. Background**

Comment: The first paragraph’s background is too lengthy; simplify it. Expand the significance of the research in the second paragraph.

Response:

We have already streamlined the first paragraph (see the Introduction, pages 3, lines 53-69). The significance of the research has been added in the Preface (page 4, lines 84-88).

**2. Literature review and hypotheses establishment**

Comment: Increase “Literature review and hypotheses establishment”.

Response:

We have added “Literature review and hypotheses establishment”(page 4-8, lines 89-174).

**3. Table 1: Physical exercise**

Comment: In Table 1, the first four variables are mean values, but the fifth variable, Physical exercise, seems to be a sum, whether its standard deviation is also a problem, please check the data carefully.

Response:

We used the Chinese version of the Physical Activity Rating Scale-3 (PARS-3) developed by Liang Qingde to assess physical exercise. The scale included four items rated on a 1–5 scale. Exercise volume was calculated as:

Exercise Volume=Intensity×(Time−1)×Frequency

Exercise volume categories were defined as follows:

Small exercise volume (≤19 points)

Moderate exercise volume (20–42 points)

Large exercise volume (≥43 points)

Descriptive statistics for these categories are presented in Table 1.

**4. Moderating effect and Mediating effect**

Comment: The title of the author's article discusses “moderating effect”, but why does Table 2 show “mediating effect”? Please carefully identify these two concepts to avoid confusion.

Response:

To avoid ambiguity, we modify the title to: Social Support and Physical Exercise: The Mediating Role of Achievement Emotions, and the Moderating Role of Exercise Motivation and Feedback Literacy. We draw a diagram of the mediation or moderation model (page 8, lines 174).

**5. References**

Comment: Also, please reconfirm the format of the references section, for example, some citations have all capitalized authors and some have not; and is the 9th reference incomplete?

Response:

We have modified the capitalization of the references (page 22-28) and corrected the incorrect reference in item 9 (page 23, lines 469-470).

**Responses to Reviewer #3**

**1. Background**

Comment: Lack of Clear Research Motivation and Theoretical Context.

Response:

We have added “Literature review and hypotheses establishment”(page 4-8, lines 89-174).

**2. Potential Misinterpretation of Correlation**

Comment: The phrase “a significant positive correlation between them” could be misinterpreted by readers as implying causation. I suggest revising this to “a significant positive association between them” to more accurately reflect the nature of cross-sectional research and avoid suggesting a causal relationship.

Response:

We have modified "a significant positive correlation between them" to "a significant positive association between them"(page 12, lines 249-250).

**3. Missing Information on Sampling Process (L178-188)**

Comment: This section lacks details on the sample selection process, including the timing of questionnaire administration, the sampling procedure, the calculation of the minimum sample size, and the specific distribution of the sample.

Response:

The above content is reflected in "Participants" (page 8-9, lines 178-195). We have also added the calculation method of the minimum sample size (page 9, lines 188-189).

**4. Lack of Discussion on Measurement Tool Limitations**

Comment: The limitations of the measurement tool are not addressed. The text does not mention the limitations of the PARS-3 scale in terms of its scope of measurement, nor does it discuss the potential impact of these limitations on the study’s results.

Response:

We added the limitations of the Physical Exercise Rating Scale and its impact on the research results (page 16-17, lines 313-318).

**5. Ambiguity between “Physical Exercise” and “Physical Activity” in**

**Discussion**

Comment: The terms “physical exercise” and “physical activity” are used interchangeably in the discussion. While these terms can be used synonymously in some contexts, it is important to clearly distinguish between them, particularly when selecting measurement tools, to ensure the accuracy of the study.

Response:

When we asked the relevant translation agency to translate, we confused the concepts of "physical exercise" and "physical activity". We have unified the concept of the full text (physical exercise).

6. **Lack of Discussion on Theoretical Adaptation to Chinese Context**

**(L275-291)**

Comment: The manuscript mentions social support theory and expectancy-value theory but does not discuss their applicability within the Chinese cultural context. The influence of social support and motivation on affect may differ across cultures, and this warrants further discussion.

Response:

We have added a discussion in the context of Chinese culture (page18-19, lines 351-353,357-359,369-372).

7. **Unclear Definition and Measurement of “Feedback Literacy”**

Comment: The concept of “feedback literacy” is mentioned but not clearly defined or operationalized. As a relatively new concept, it requires further elaboration on its specific meaning and measurement within the context of physical exercise.

Response: We have added the definition of feedback literacy (page 7, lines 145-146).

8. **Lack of Discussion on Cultural Adaptation of Theory and Scales**

**(L322-332)**

Comment: The cultural adaptation of the theories and scales used in the Chinese context is not discussed. The impact of social support, motivation, and affect on physical exercise may vary across different cultural backgrounds.

Response: We have added a discussion on "The cultural adaptation of the theories and scales used in the Chinese context"(page 21, lines 411-414).

9. **Standardized Abstract**

Comment: I recommend using a standardized abstract format, including sections on Purpose, Methods, Results, and Conclusions.

Response: We have completed the modification of a standardized abstract format (page 2, lines 27,33,36,45).

We sincerely appreciate your time and consideration. Please contact us if further revisions are required.

Best regards,

Zhang Hui

On behalf of all authors
